# Nurses' Trials.—Weariness and Prayer

**Published:** 1887-02-12

**Authors:** 


### WORDS OF CONSOLATION.

[Communications for this column should be addressed, "Chaplain," care of the Editor of THE HOSPITAL, who will gratefully welcome any suggestions or inquiries from matrons, nurses, and the staff generally.]

### XX.—Nurses' Trials—Weariness and Prayer.

Weariness is a trial in itself to anyone when it becomes constant. But it is harder still for the spiritually minded to endure, or for such as have been brought up from childhood to value religious privileges. It is difficult for a nurse to pray with regularity. Her time is necessarily much broken up. A call to some necessitous case may arise just as she is about to offer her morning or evening devotions; and, when this kind of thing happens repeatedly, the temptation to put those devotions aside is very great. It is to be feared that many persons who enter states of life in which they are subject to be called away at any hour at a moment's notice, gradually leave off praying; or, at all events, they cease from any regular habits of prayer, from simply finding it impossible to say their prayers as they have been wont to in childhood. The old accustomed times for prayer are broken through to begin with, and they do not value prayer sufficiently to think of making other times for it instead. Now, weariness of body is one of the greatest temptations in such cases. You have perhaps found yourself too tired to pray when you would; or, you have been interfered with almost in the act of kneeling down; so the devotions had to be sacrificed; but you did not think of praying *when you could at another time instead.* We are most of us brought up to say our prayers; so the habit is formed. But sooner or later God tries whether we really value prayer sufficiently to make a point of it in spite of hindrances. Those who have learnt not merely to say their prayers, but to pray them, know and feel that they cannot do without them. They have found prayer as essential to the soul as the very air they breathe is to the body. If they cannot hold communion with their Heavenly Father at one time, they will insist upon it at another. We scarcely need urge such good Christians as these to do what the Holy Spirit within them is ever moving them to. But these are the few. To others inclined to fall into neglect under temptation through weariness or work, we would simply say—"You are now in the thick of the fight. Your vocation brings you into the very heat of the battle with sickness, suffering, ay, and with sin. Therefore, you can less afford to put off your spiritual armour than at any previous period of your life. You complain that your once formed habits of devotion are sadly interrupted. Then make time for them when you can. If you have no time for morning prayers upon getting up, surely the first few minutes in the day you can find for yourself ought to be given to them. If you are too tired to collect your thoughts when you retire to rest, make a point of saying your evening prayers an hour or two earlier as opportunity occurs. Anything sooner than sacrifice the habit. Insist on making time, to begin with; and you will gradually realize the vast importance of this to yourself and to those whom you watch over. The inward life must on no account be suffered to decay because the outward is so busy. The very business and weariness of the outward life provide, not the excuse for neglecting, but the strongest reason for maintaining the spirit of devotion. "Come unto Me ye weary and heavy laden, and I will give you rest," and again, "Pray without ceasing," were neither of them words spoken to people who had a great deal of leisure-time for devotion. How many of us leading busy lives have yet to learn that frequent converse with God is the way to make the yoke easy and the burden light!

